# Differential Hippocampal Expression of BDNF Isoforms and Their Receptors Under Diverse Configurations of the Serotonergic System in a Mice Model of Increased Neuronal Survival

**DOI:** 10.3389/fncel.2019.00384

**Published:** 2019-08-21

**Authors:** Rocío Beatriz Foltran, Karen Melany Stefani, Antonela Bonafina, Agustina Resasco, Silvina Laura Diaz

**Affiliations:** Instituto de Biología Celular y Neurociencia Prof. E. De Robertis, CONICET – Universidad de Buenos Aires, Buenos Aires, Argentina

**Keywords:** BDNF signaling pathway, serotonin depletion, 5-HT_1A_ receptor, mice model, newborn neurons survival, pattern separation

## Abstract

Neurotrophic factors are relevant regulators of the neurogenic process at different levels. In particular, the brain-derived neurotrophic factor, BDNF, is highly expressed in the hippocampus (HC) of rodents and participates in the control of neuronal proliferation, and survival in the dentate gyrus (DG). Likewise, serotonin is also involved in the regulation of neurogenesis, though its role is apparently more complex. Indeed, both enhancement of serotonin neurotransmission as well as serotonin depletion, paradoxically increase neuronal survival in the HC of mice. In this study, we analyzed the protein expression of the BDNF isoforms, i.e., pro- and mature-BDNF, and their respective receptors p75 and TrkB, in the HC of mice chronically treated with para-chloro-phenyl-alanine (PCPA), an inhibitor of serotonin synthesis. The same analysis was conducted in hyposerotonergic mice with concomitant administration of the 5-HT_1__*A*_ receptor agonist, 8-Hydroxy-2-(di-n- propylamino) tetralin (8-OH-DPAT). Increased expression of p75 receptor with decreased expression of pro-BDNF was observed after chronic PCPA. Seven-day treatment with 8-OH-DPAT reestablished the expression of pro-BDNF modified by PCPA, and induced an increase in the expression of p75 receptor. It has been demonstrated that PCPA-treated mice have higher number of immature neurons in the HC. Given that immature neurons participate in the pattern separation process, the object pattern separation test was conducted. A better performance of hyposerotonergic mice was not confirmed in this assay. Altogether, our results show that molecules in the BDNF signaling pathway are differentially expressed under diverse configurations of the serotonergic system, allowing for fine-tuning of the neurogenic process.

## Introduction

With the extension of life expectancy, several pathologies affecting the central nervous system have gained greater visibility. In this context, disentangling the etiopathogenesis of neurodegenerative diseases and seeking for more effective therapies represent key challenges. Strategies to replace lost neurons because of neurodegenerative processes are focused on two types of interventions: on the one hand, transplantation of exogenous nerve cells and, on the other, the use of the endogenous neural niches still active in the adult nervous system (Boda et al., 2017). Indeed, neurogenic potential has been specifically characterized in the hippocampus (HC) and the subventricular zone of adult mammal brains (Gage, 2000). However, a thorough knowledge of the molecular mechanisms controlling neurogenesis and its possible manipulation in pathological conditions is required in view of its therapeutic use. In this sense, neurotrophic factors have recently emerged as regulators of the neurogenic process at different levels (see review in Vilar and Mira, 2016). In particular, the brain-derived neurotrophic factor, BDNF, is highly expressed in the HC of rodents (Aid et al., 2007), and its role in the regulation of the proliferation and survival of newborn neurons in the dentate gyrus (DG) has been extensively studied (see review in Foltran and Diaz, 2016). BDNF is secreted as pro-BDNF, a proneurotrophin which can be converted to mature BDNF (mBDNF). Pro-BDNF acting through the p75 receptor is responsible for pro-apoptotic actions, whereas mBDNF-TrkB receptors complex mediates pro-neurogenic effects. Globally, the actions of both BDNF isoforms contribute to the regulation of neuronal homeostasis.

At the beginning of this century, an original report showed that chronic administration of serotonin selective reuptake inhibitors antidepressants is able to induce neurogenesis in the HC of adult rats (Malberg et al., 2000). Since this pivotal discovery, the neurogenic actions of serotonergic drugs were extensively proved in the adult HC of mice and even in humans (see review in Kempermann et al., 2018). However, the specific role of serotonin is still unclear, since increased neuronal survival is also described in the HC of diverse mice models of constitutive (Diaz et al., 2013; Karabeg et al., 2013; Sachs et al., 2013; Song et al., 2017) as well as induced (Jia et al., 2014; Song et al., 2016) serotonin depletion. Particularly, we have previously reported increased survival of 1 week- and 4 week- old newborn neurons in the DG of mice chronically treated with PCPA during 5 and 8 weeks, respectively (Diaz et al., 2013). These paradoxical effects, i.e., supernumerary newborn neurons in the DG of mice with either increased or decreased serotonergic neurotransmission, could be partially explained by the participation of one or several of the serotonin receptors subtypes. Indeed, stimulation of 5-HT_1__*A*_ receptor for 7 days was enough to reestablish the increased survival of 1-week-old neurons shown in hyposerotonergic mice, induced either by genetic, or pharmacological ablation (Diaz et al., 2013).

Immature neurons in the DG appear to be responsible for enhanced pattern separation, i.e., the ability to discriminate between two very similar but different spatial contexts (Sahay et al., 2011; Nakashiba et al., 2012). Nevertheless, we have shown that hyposerotonergic Pet1^–/–^ mice, displaying increased number of immature neurons in the DG, and have a normal performance in contextual fear-discrimination learning (Diaz et al., 2013). A recently developed test, called object pattern separation (van Hagen et al., 2015), provides the opportunity to measure both deterioration, and improvement in the ability of pattern separation. It is based on the natural tendency of rodents to explore an object that is new to them, so they do not have to be trained for a special skill and it is not highly stressful to the animals. Likewise, its highest resolution allows finding subtle differences, which may shed light in the search for the specific role of immature neurons in hyposerotonergic mice models.

We analyze here the protein expression of the BDNF signaling pathway in the HC of hyposerotonergic mice, revealing changes in the expression of p75 receptor and pro-BDNF. Likewise, 7-day treatment with a 5-HT_1__*A*_ receptor agonist to these mice partially reversed changes induced by serotonin depletion. Finally, hyposerotonergic mice did not performed better than control mice in the objet pattern separation test.

## Materials and Methods

### Animals

Studies were performed on 102 male, C57BL/6 elite mice purchased at the Instituto de Medicina Experimental, Academia Nacional de Medicina, Buenos Aires, Argentina. Experiments on animals were conducted according to local regulations and were approved by the Institutional Ethical Committee (UBA-FMED, Resol. 2016/1637 and 2019/297). Three-four week-old mice, bred in barrier-conditions to maintain an SPF status, were transported in environmentally controlled conditions to our institute’s animal facility. After arrival, mice were housed in 1284L Eurostandard Type II Long (365 mm × 207 mm × 140 mm) Tecniplast microisolator cages with filter tops (five to seven animals per cage), with autoclavated aspen shavings as bedding and tissue paper as nesting material. Mice were maintained under controlled conditions, i.e., 22 ± 2°C room temperature, 60% relative humidity, 12–12 h light–dark cycle (lights on at 8 a.m.), pelleted food for rodents (Cooperación) and water *ad libitum*. Cages were changed twice a week. A period of acclimation of 2 weeks was left before the beginning of experiments, and therefore, mice were 5–6 week-old when treatments begun.

### Serotonin Depletion Protocol

Serotonin depletion in mice was induced by oral administration of para-chloro-phenyl-alanine (PCPA), an inhibitor of the rate limiting enzime tryptophane hydroxylase, during 8 weeks, as previously described (Foltran et al., 2019). Briefly, PCPA was suspended in a 0,5% carboxymethyl cellulose solution and mixed in gelatin palatable cubes. Mice were divided into two groups. One group received cubes with PCPA at an estimated dose of 500 mg/kg on days 1 and 2, and 250 mg/kg PCPA for the rest of the treatment (69 mg/cube, and 34.5 mg/cube respectively, per cage with 6 mice). The control group received similar cubes with vehicle. A PCPA or vehicle containing cube was given per cage, every day between 2 p.m. and 5 p.m. Serotonin depletion in the HC (75%) and cortex (60%) of mice was confirmed by HPLC as soon as 10 days after the beginning of PCPA administration (Foltran et al., 2019).

### Experiment 1: Expression of the BDNF Signaling Pathway

Chronic PCPA treatment during 8 weeks increases survival of 4 week-old neurons in the HC (Diaz et al., 2013). To study the potential involvement of the BDNF signaling pathway in these neurogenic effects, the protein expression of BDNF isoforms and their receptors was studied by western blotting. After 8 week of PCPA (*n* = 6) or vehicle (*n* = 5) administration, mice were killed by cervical dislocation and both HC were obtained. Tissue was homogenized with 250 μl of RIPA buffer along with protease inhibitors (150 mM NaCl, 1% NP-40, 0.5% Sodium deoxycholate, 0.1% SDS, 50 mM Tris) and centrifuged at 4°C for 30 min at 13000 (r/min). The supernatants were recovered and proteins levels were quantified by the Bradford protein assay. Samples (50 μgr in 5× loading buffer) were then loaded into SDS-PAGE gels (12 or 15%) and transferred onto nitrocellulose membranes using the Mini-PROTEAN^®^ Tetra System (BIO-RAD) for 1 h.

### Western Blotting

Membranes were incubated for 1 h with blocking solution (5% milk in TBST) and then probed overnight at 4°C with mouse anti-BDNF (1:2000; Icosagen; 327-100 clone 3C11), rabbit anti-p75 (1:700; Alomone Labs; ANT-007), rabbit anti-TrkB (1:700; Alomone Labs; ANT-019), and rabbit anti-proBDNF (1:250; Abcam; ab72440) in TBST. β-III Tubulin was used as a loading control (1:2500; R&D Systems). Binding of primary antibodies was visualized with anti-mouse HRP-conjugated secondary antibody (1:3000; BIO-RAD) or anti-rabbit HRP-conjugated secondary antibody (1:3000; BIO-RAD). Membranes were developed using the ECL Plus Western blotting substrate (Thermo Fisher Scientific) for chemifluorescence with the Storm^®^ Molecular Imager. Densitometry was carried out using ImageJ software (Schneider et al., 2012). The signal of each protein is expressed after subtraction of background signal and related to tubulin signal.

### Experiment 2: Chronic Treatment With the 5-HT_1__*A*_ Agonist, 8-OH-DPAT

We have previously reported that chronic PCPA induced increased survival of 1 week- and 4 week-old newborn neurons corresponding to 5 and 8 weeks of PCPA administration, respectively (Diaz et al., 2013). Also we have shown that stimulation of the 5-HT_1__*A*_ receptor for 7 days is able to reestablish the basal level of survival of 1 week-old newborn neurons in the HC of PCPA-chronically treated mice (Diaz et al., 2013). To study the role of this serotonin receptor on the BDNF signaling pathway of hyposerotonergic mice, the 5-HT_1__*A*_ receptor agonist 8-Hydroxy-2-(di-n- propylamino)tetralin (8-OH-DPAT) was administered to a group of PCPA- (*n* = 11) or vehicle- (*n* = 12) treated mice. Mice received palatable cubes with PCPA or vehicle for 4 weeks, as explained above. On the 5th week, animals continued receiving PCPA or vehicle, and simultaneously received two daily intraperitoneal (i.p.) injections of 8-OH-DPAT at 0.5 mg/kg or vehicle (NaCl 0.9%) for 7 days as previously described (Diaz et al., 2013). Mice were killed by cervical dislocation on the day of the last injection and HC were collected. Western blotting was performed as already described.

### Experiment 3: Object Pattern Separation (OPS) Test in Hyposerotonergic Mice

For an initial set up of the object pattern separation (OPS) test in our experimental conditions, the protocol described by van Hagen et al. (2015), was followed with few modifications. A 27 cm diameter-round open field with red walls was employed (Figure 3A). This arena was placed inside a bigger black square open field with visual cues fixed in each wall. Briefly, mice were divided into 5 groups (*n* = 8/experimental group) and habituated for 3 days to the open field without any objects. They were placed in the arena and left for 10 min to explore. Mice were then tested 48 hs after the last habituation session. First, in a pre-test session, they were presented two identical objects placed in two symmetrical spots in the center of the arena. The objects were caramel color bottles of 7 cm of height filled with a dark solution so that mice were not able to move them. Animals were left 4 min to explore. The test session was performed 1 h later, where one of the objects was placed in a novel location inside the arena 2, 4, 6, or 8 cm away from the initial location (positions 2, 3, 4, and 5, respectively). Position 1 meant no change in any object. Each group was assigned a different position. Mice were left again 4 min to explore. Time spent exploring each object in both sessions was recorded. Exploration was defined as directing the nose toward the object at a distance of no more than 2 cm and/or touching the object with the nose. Sitting on the object was not considered as exploratory behavior. Mice that spent less than 10 s in total exploratory time in the pre-test or test were eliminated from the data, since it has been shown that animals require at least 10 s of object interaction for reliable object discrimination (şik et al., 2003; Akkerman et al., 2012). Exploration time was measured manually in a computer with the Solomon Coder program. A discriminatory index was calculated, as “(time spent exploring the moved object – time spent exploring the stationary object)/total exploratory time.” According to the results of the set up, the position 3 was chosen to test treated mice.

To study the effects of PCPA treatment on the process of pattern separation, mice were divided in two groups (*n* = 13–15), and received a palatable cube containing either PCPA or vehicle during 8 weeks, as described above. PCPA was administered as a tool to increase the survival of newborn neurons in the HC (Diaz et al., 2013). The OPS test was conducted as described above, on the 7th week of PCPA or vehicle treatment. All habituation and test sessions were recorded, and videos were analyzed to measure exploratory activity. Distance moved by mice in the habituation videos were used to quantify locomotor activity, via EthoVision XT 14. Time spent exploring each object in both, the pre-test or test sessions, was quantified to calculate the discriminatory index as already explained.

### Chemical Substances

PCPA (4-Chloro-DL-phenylalanine): Sigma, C6506; 8-OH-DPAT (8-Hydroxy-2-(dipropylamino) tetralin hydrobromide): Sigma, H8520.

### Statistical Analysis

All data were checked to verify whether normality and homoscedasticity assumptions were met. Different treatments in Experiment 1 were analyzed using Student’s *t*-test. Results in Experiment 2 were analyzed by a two-way analysis of variance (ANOVA), with hyposerotonergic condition, and DPAT treatment as factors. Tukey’s test was used for *post hoc* comparisons. Simple effects tests were performed to analyze differences inside each condition. The OPS was analyzed using the one-sample t-statistics in order to assess whether the discrimination index, for each experimental condition separately, differed significantly from 0. In all cases, *P* < 0.05 was considered statistically significant. A summary of tests employed and statistics is presented in Table 1.

**TABLE 1:** *P* values.

**Paradigm or assay**	**Parameter measured**	**Statistical test**	**Comparison**		**Statistics**	**Degree of freedom**	***P* value**	**Figure**
Western blot	mBDNF signal relative to tubulin	Student’s *t* test	Treatment		*T* = 1.056	21	0,3032	1A
Western blot	TrkB signal relative to tubulin	Student’s *t* test	Treatment		*T* = 0.9418	21	0,3570	1B
Western blot	proBDNF signal relative to tubulin	Student’s *t* test	Treatment		*T* = 1.577	18	0,1304	1C
Western blot	P75 signal relative to tubulin	Student’s *t* test	Treatment		*T* = 2.157	21	0,0427	1D
Western blot	mBDNF signal relative to tubulin	Two-way ANOVA	Interaction		*F* = 3,38	1	0,0817	2A
			Factor food		*F* = 13,19	1	0,0018	
			Factor injection		*F* = 1,3^*E–*03^	1	0,9714	
		Tukey post-test	Control – DPAT				A	
			Control – NaCl				AB	
			PCPA – NaCl				AB	
			PCPA – DPAT				B	
		Simple effects	Inside food	Ctrl	*F* = 5,73	1	0,0403	
				PCPA	*F* = 1,11	1	0,3161	
			Inside Inject	NaCl	*F* = 1,17	1	0,3039	
				DPAT	*F* = 27,71	1	0,0005	
Western blot	TrkB signal relative to tubulin	Two-way ANOVA	Interaction		*F* = 5,34	1	0,0322	2B
			Factor Food		*F* = 1,45	1	0,2433	
			Factor Injection		*F* = 19,36	1	0,0003	
		Tukey post-test	Control – DPAT				A	
			Control – NaCl				A	
			PCPA – NaCl				AB	
			PCPA – DPAT				B	
		Simple effects	Inside food	Ctrl	*F* = 29,03	1	0,0004	
				PCPA	*F* = 1,86	1	0,2027	
			Inside inject	NaCl	*F* = 4,23	1	0,0668	
				DPAT	*F* = 1,44	1	0,2614	
Western blot	proBDNF signal relative to tubulin	Two-way ANOVA	Interaction		*F* = 4,63	1	0,0453	2C
			Factor food		*F* = 0,43	1	0,5214	
			Factor injection		*F* = 11,59	1	0,0032	
		Tukey post-test	Control – DPAT				A	
			Control – NaCl				AB	
			PCPA – NaCl				B	
			PCPA – DPAT				B	
		Simple effects	Inside food	Ctrl	*F* = 0,79	1	0,3973	
				PCPA	*F* = 15,36	1	0,0035	
			Inside inject	NaCl	*F* = 11,25	1	0,0085	
				DPAT	F = 0,68	1	0,4314	
Western blot	P75 signal relative to tubulin	Two-way ANOVA	Interaction		*F* = 5,63	1	0,0284	2D
			Factor food		*F* = 22,70	1	0,0001	
			Factor Injection		*F* = 6,79	1	0,0174	
		Tukey post-test	Control – DPAT				A	
			Control – NaCl				A	
			PCPA – NaCl				A	
			PCPA – DPAT				B	
		Simple effects	Inside food	Ctrl	*F* = 0,39	1	0,5469	
				PCPA	*F* = 7,07	1	0,0239	
			Inside inject	NaCl	*F* = 3,45	1	0,0931	
				DPAT	*F* = 21,28	1	0,0013	
Object pattern separation	Discrimination index	One sample *t* test	P1 vs. 0		*T* = −0,03	–	0,9810	3B
			P2 vs. 0		*T* = 0,30	–	0,7706	
			P3 vs. 0		*T* = 1,77	–	0,1203	
			P4 vs. 0		*T* = 1,03	–	0,3618	
			P5 vs. 0		*T* = 3,82	–	0,0088	
Object pattern separation	discrimination index	One-way ANOVA	Between positions		*F* = 1,81	4	0,1544	3B
Object pattern separation	Habituation	Two-way ANOVA	Interaction		*F* = 0,14	2	0,8694	3C
			Factor treatment		*F* = 1,05	1	0,3131	
			Factor Day		*F* = 17,98	2	<0,0001	
		Tukey post-test	Day 1 vs. Day 2				<0,05	
			Day 1 vs. Day 3				<0,05	
			Day 2 vs. Day 3				ns	
Object pattern separation	Discrimination index	One sample *t* test	Control vs. 0		*T* = 2,18	–	0,0435	3D
			PCPA vs. 0		*T* = 0,43	–	0,6736	

## Results

### Experiment 1: Expression of the BDNF Signaling Pathway

**FIGURE 1:**
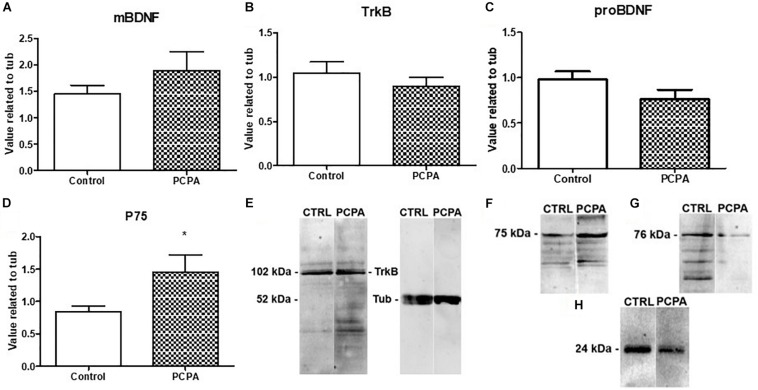
Protein expression of molecules from the BDNF signaling pathway determined by Western Blot and representative membranes. **(A)** mBDNF. **(B)** TrkB. **(C)** proBDNF. **(D)** p75 in hyposerotonergic (PCPA-treated) or control (vehicle-treated) mice for 4 weeks. **(E–H)** Representative membrane showing signal for TrkB, p75, pro-BDNF, and mBDNF in Control and PCPA-treatd mice. Data are expressed as mean ± S.E.M., *n* = 11 (control) and 12 (PCPA). ^∗^*p* < 0.05.

Protein-expression of BDNF isoforms and their receptors was analyzed in the HC of 8-week PCPA- or vehicle-treated mice (Figures 1A–H; see statistics in Table 1). Whereas no significant differences were found between experimental groups for mBDNF, its receptor TrkB, and proBDNF, expression of the proBDNF receptor, p75, was significantly increased (*p* = 0.0427) in PCPA-treated mice.

### Experiment 2: Chronic Treatment With the 5-HT_1__*A*_ Agonist, 8-OH-DPAT

**FIGURE 2:**
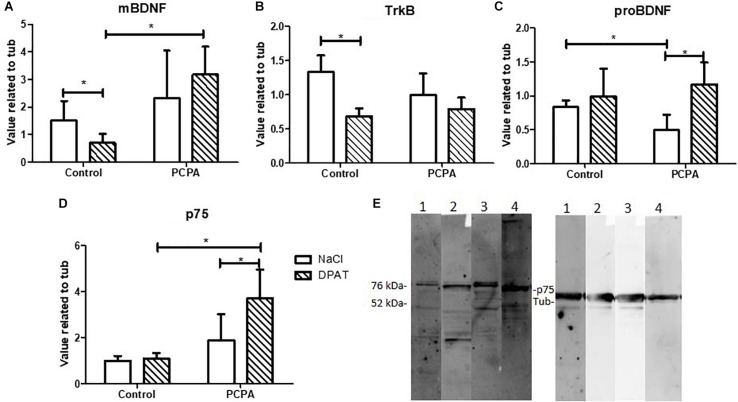
Effect of 8-OH-DPAT on the protein expression of molecules from the BDNF signaling pathway in the HC, determined by Western Blot. **(A)** mBDNF. **(B)**TrkB. **(C)** proBDNF. **(D)** p75 in hyposerotonergic (PCPA-treated) or control (vehicle-treated) mice receiving the 5-HT_1__*A*_ agonist 8-OH-DPAT (striped bars) or NaCl (white bars). **(E)** Representative membrane showing signal for p75 (75 kDa) and tubuline (52 kDa). 1: Control-NaCl. 2: Control-DPAT. 3: PCPA-NaCl. 4: PCPA-DPAT. Data are expressed as mean ± S.E.M., *n* = 6 per experimental group.^∗^*p* < 0.05.

Protein expression of the BDNF isoforms and their receptors was analyzed in the HC of PCPA- and vehicle- chronically treated mice receiving the 5-HT_1__*A*_ agonist DPAT or NaCl 0.9% (Figures 2A–E). Statistical analysis showed a significant interaction between hyposerotonergic condition and DPAT treatment for TrkB, proBDNF, and p75 receptor (for statistics, see Table 1). Five weeks of PCPA administration induced a decreased expression of proBDNF (*p* = 0.0085), and a tendency to increased expression of p75 receptor (*p* = 0.0931), without significantly modifying the expression of mBDNF or TrkB receptor. One week administration of DPAT in vehicle-treated mice induced a significant decrease in the expression of mBDNF (*p* = 0.0403) and TrkB receptor (*p* = 0.0004), without significantly affecting the levels of proBDNF or p75 receptor. Finally, administration of DPAT to PCPA-treated mice induced a significant increase in the expression of proBDNF (*p* = 0.0035) and p75 receptor (*p* = 0.0239), without significantly modifying the levels of mBDNF and TrkB receptor.

### Experiment 3: Object Pattern Separation (OPS) Test

**FIGURE 3:**
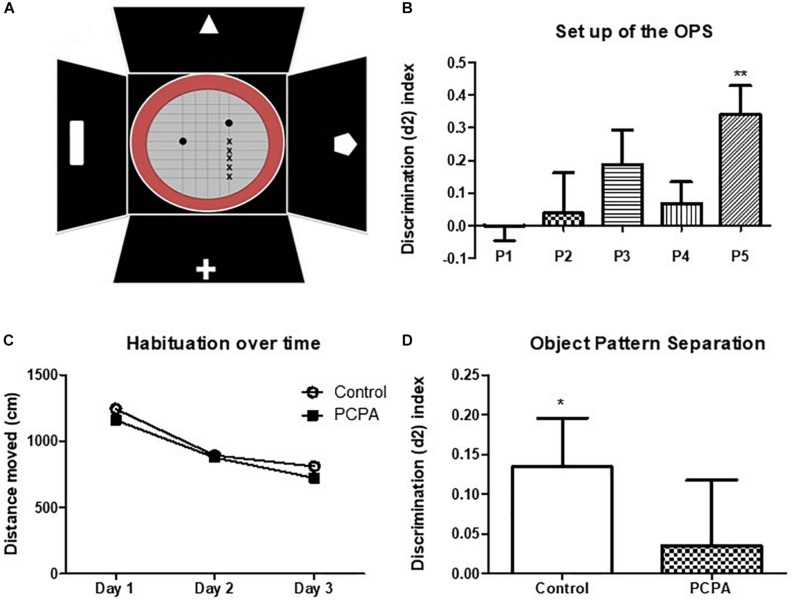
Object pattern separation (OPS) test. **(A)** Scheme of the arena and objects used for the test. The *x* represents the different possible positions for the moved object. Full circles represent the bottles used as objects. **(B)** Discrimination index for the different new positions for the moved object in naive mice. **(C)** Locomotor activity in an open field along 3 days. **(D)** Discrimination index in the OPS of mice chronically treated with PCPA or vehicle (control) for 8 weeks. Data are expressed as mean ± SEM., *n* = 13 (Control) and 15 (PCPA).^∗^*p* < 0.05; ^∗∗^*p* < 0.01.

In the set up, each mouse was assigned to a different position for the moved object, identified as P1, P2, P3, P4, or P5 (Figure 3A). As the objects were more separated from the original position, the discrimination index became higher and increasingly different from 0 (Figure 3B). Although results at P4 were unexpected, P5 resulted in an index significantly different from 0 (*p* = 0.0088), whereas P3 showed a tendency for significance (*p* = 0.1203). Altogether, as naïve mice are barely able to discriminate positions in P3, the performance in this position can be improved, which would result in a higher index and possibly, a *p* value <0.05. If mice were tested in P5, where the discrimination index is high, a difference between treated and control mice would not be seen, as all of them are expected to perform well.

To study the role of newborn cells in the HC of mice treated with PCPA or vehicle, animals were assayed in the OPS at P3, given that subtle differences, i.e., a mild better performance, between experimental groups were expected. Mice were habituated to the open field for 3 consecutive days, for 10 min each day. All mice got habituated to the arena (Figure 3C), with less distance traveled on each passing day (*p* < 0.05). No significant difference in locomotor activity was seen between mice treated with PCPA and vehicle. After a resting day, mice were presented with two identical bottles for 4 min at P1. One hour later, one of the bottles was moved to P3 and the animals were left another 4 min to explore. Five mice were eliminated from the data because they did not meet the criteria for minimal exploration time of 10 s. A discrimination index was calculated, as previously explained (Figure 3D). Whereas the index in control mice was significantly different from 0 (*p* = 0.0435), PCPA-treated mice showed a high *p* value (*p* = 0.6736).

## Discussion

Previous reports on hyposerotonergic mice demonstrated increased survival of the newborn neurons generated in the adult HC, with unchanged cell proliferation. The results presented herein support the hypothesis about an interplay between the BDNF signaling pathway in the HC and the serotonergic system that could participate in the regulation of the survival of newborn neurons. On the other hand, in our experimental conditions, a facilitating role for supernumerary immature neurons on pattern separation assays could not be confirmed.

Interestingly, a recent study, found increased mBDNF expression in the prefrontal cortex of 1 month-old Tph2-deficient rats (Brivio et al., 2018). Although this result observed in serotonin-deficient rats was not reproduced in our mice model, it is not completely unexpected. Indeed, several studies have analyzed the effects of serotonin depletion on the neurogenic process in rats and mice, revealing a clear dichotomy: whereas lack of serotonin induces decreased survival of newborn neurons in the DG of rats (Brezun and Daszuta, 1999, 2000; Banasr et al., 2001; Jha et al., 2006; Kohl et al., 2016), the opposite effect has been demonstrated in serotonin-depleted mice (Diaz et al., 2013; Karabeg et al., 2013; Jia et al., 2014; Song et al., 2016, 2017). Therefore, caution is required when comparing neurogenic effects in these two rodent species. Indeed, there are few reports conducted in hyposerotonergic mice. In the present study, a mice model in which serotonin depletion was pharmacologically induced, after inhibition of the Tph rate-limiting enzyme, was employed. This pharmacological model, allows us to specifically target the process of adult neurogenesis, in opposition to transgenic mice like Pet1^–/–^ (Diaz et al., 2013), Tph2^–/–^, SERT^–/–^ (Kronenberg et al., 2016) mice in which constitutive depletion of serotonin affects as well the process of developmental neurogenesis that occurs during the first 2 weeks of life. In our experimental conditions, an increased protein expression of the p75 receptor was detected in the HC of mice after 4 and 5 weeks of PCPA administration. In addition, after 5 weeks of PCPA treatment, reduced protein expression of the p75 receptor ligand pro-BDNF was also evident. Since pro-BDNF is known to facilitate cell death (Barker, 2004), the decreased levels revealed herein in the HC of hyposerotonergic mice are in line with the increased cell survival previously reported in mice treated with PCPA for 8 weeks (Diaz et al., 2013). On the contrary, we did not observe any change in the expression of mBDNF and its receptor TrkB after chronic PCPA. These observations are in agreement with a recent article conducted in mice in which serotonin synthesis disruption was induced during adulthood (Pratelli et al., 2017). Indeed, Tph2 was specifically knocked-out at post-natal day 60 and hippocampal BDNF and TrkB receptor expression was unchanged compared to wild type mice. On the contrary, studies on the HC of mice with constitutive serotonin depletion showed an increase of BDNF mRNA (Migliarini et al., 2013) as well as of BDNF protein levels (Kronenberg et al., 2016). All in all, these results suggest that lack of serotonin induces dichotomic responses in the BDNF signaling pathway depending on the time of onset of serotonin depletion.

Stimulation of 5-HT_1__*A*_ receptors during 4 weeks was described to increase cell proliferation (Santarelli et al., 2003). However, shorter periods of stimulation, i.e., 1–2 week, did not induced neurogenic effects (Banasr et al., 2004; Huang and Herbert, 2005; Klempin et al., 2010; Diaz et al., 2013). Here, in our study, 1-week administration of the 5-HT_1__*A*_ agonist promoted a decreased protein expression of mBDNF and TrkB receptor in the HC of control mice. To our knowledge, the only data published in mice so far, concerned animals receiving DPAT from postnatal day 1 to 21, yielding a trend to decreased expression of BDNF mRNA in the HC (Ishikawa and Shiga, 2017). Interestingly, in the case of hyposerotonergic mice, 1-week stimulation of the 5-HT_1__*A*_ receptor did not significantly affect the mBDNF/TrkB pathway, but induced an increased protein expression of proBDNF and p75 receptor. Considering the pro-apoptotic properties reported for this complex (Barker, 2004), its enhanced expression could be playing a role in the reestablishment of cell survival in DPAT-treated hyposerotonergic mice (Diaz et al., 2013). All in all, our results suggest that the BDNF signaling pathway is differentially affected according to the serotonergic neurotransmission context.

The object pattern separation task allows the detection of subtle differences in mice performance (van Hagen et al., 2015). Surprisingly, PCPA-treated mice failed to get an index significantly different from 0, as it was the case for control mice. Although PCPA-treated mice have higher number of immature neurons in the HC (Diaz et al., 2013), this result was not completely unexpected, and given that we have previously found no improvement in the performance of hyposerotonergic mice in the contextual fear discrimination learning test (Diaz et al., 2013). As serotonin is involved in several different processes in the brain, reduced serotonin levels may impair cognitive abilities required to solve this kind of tests, independently of the number of immature neurons. It is interesting to mention that when setting up the OPS test, we were able to reproduce the discrimination curve reported in the original work (van Hagen et al., 2015), except for the position 4, where we could not get a discrimination index intermediate between P3 and P5. However, this unforeseen fact do not appear to interfere with the obtained results.

The observations made in our experimental conditions shed light on the role of the BDNF isoforms in the regulation of the neurogenic process that takes place in the HC of adult mice, under different configurations of the serotonergic system. This knowledge is key to propose new therapeutic targets for the development of more efficacious drugs.

## Data Availability

All datasets generated for this study are included in the manuscript and/or the supplementary files.

## Ethics Statement

Experiments on animals were conducted according to the local regulations and were approved by the Institutional Ethical Committee (UBA-FMED, Resol. 2016/1637 and 2019/297).

## Author Contributions

RF designed the study, conducted the experiments, and wrote the manuscript. KS, AB, and AR conducted the experiments. SD designed the study, supervised the experiments, wrote the manuscript, and provided funding.

## Conflict of Interest Statement

The authors declare that the research was conducted in the absence of any commercial or financial relationships that could be construed as a potential conflict of interest.
